# Supplementary material for: Prevalence and incidence of tuberculosis infection among healthcare workers in chest diseases hospitals, Bangladesh: Putting infection control into context
Source: PLoS One. 2023 Sep 27;18(9):e0291484. doi: 10.1371/journal.pone.0291484 (PMC10529546; doi:10.1371/journal.pone.0291484)
Supplement: S1 File — (DOCX) [file pone.0291484.s001.docx]

# Table S1: Factors associated with baseline two-step TST positivity among HCWs in four chest diseases hospitals, Bangladesh, 2013

|  | **TST positive % (n/N)** | **TST negative % (n/N)** | **OR (95% CI)** |  |
| --- | --- | --- | --- | --- |
| **Location of hospital**^i^ |  |  |  |  |
| Chittagong | 37 (12/32) | 63 (20/32) | 2.22 (0.86-5.68) |  |
| Rajshahi | 26 (12/47) | 74 (35/47) | 1.90 (1.01-3.59) |  |
| Dhaka | 17 (44/252) | 83 (208/252) | 0.78 (0.39-1.56) |  |
| Khulna | 21 (13/61) | 79 (48/61) | Reference |  |
| **Sex** |  |  |  |  |
| Male | 24 (43/181) | 76 (138/181 | 1.42 (0.87-2.32) |  |
| Female | 18 (38/211) | 82 (173/211) | Reference |  |
| **History of BCG vaccination** ^ii^ | | | | |
| Yes | 22 (67/306) | 78 (239/306) | 1.38 (0.72-2.66) |  |
| No | 17 (13/77) | 83 (64/77) | Reference |  |
| Don't know | 11 (1/9) | 89 (8/9) | 0.62 (0.07-5.35) |  |
| **Profession** | | | |  |
| Doctorsincluding interns and residents and pharmacists | 18 (7/39) | 82 (32/39) | 0.59 (0.23-1.55) |  |
| Nurse | 18 (29/161) | 82 (132/161) | 0.60 (0.31-1.13) |  |
| Admin Officer | 27 (21/78) | 73 (57/78) | Reference |  |
| Laboratory Staff | 27 (6/22) | 73 (16/22) | 1.01 (0.35-2.95) |  |
| Ancillary workers | 20 (18/92) | 80 (74/92) | 0.66 (0.32-1.35) |  |
| **Highest education completed** | | | | |
| None to Primary | 19 (7/37) | 81 (30/37) | 0.71 (0.28-1.81) |  |
| Secondary | 19 (16/86) | 81 (70/86) | 0.69 (0.34-1.41) |  |
| Higher Secondary | 20 (33/168) | 80 (135/168) | 0.74 (0.41-1.34) |  |
| Bachelor and above | 25 (25/101) | 75 (76/101) | Reference |  |
| **Years worked on pulmonary TB patient wards**^iii^ | | | | |
| <10 | 20 (41/206) | 80 (165/206) | Reference |  |
| 11-20 | 20 (16/79) | 80 (63/79) | 1.02 (0.54-1.95) |  |
| >20 | 15 (6/39) | 85 (33/39) | 0.73 (0.29-1.86) |  |
| **Hours worked on pulmonary TB wards per day^iv^** | | | | |
| ≤2 | 21 (37/173) | 79 (136/173) | Reference |  |
| >2 | 17 (26/151) | 83 (125/151) | 0.76 (0.44-1.33) |  |
| **Years of service as a healthcare worker^v^** | | | | |
| < 10 | 21 (51/248) | 79 (197/248) | Reference |  |
| 11-20 | 23 (22/94) | 77 (72/94) | 1.18 (0.67-2.08) |  |
| >20 | 16 (8/50) | 84 (42/50) | 0.74 (0.33-1.67) |  |
| **Age of starting work in years ^vi^** | |  |  |  |
| <20 | 20 (9/45) | 80 (36/45) | Reference |  |
| 20-25 | 19 (36/186) | 81 (150/186) | 0.96 (0.42-2.17) |  |
| 26-30 | 24 (29/121) | 76 (92/121) | 1.26 (0.54-2.92) |  |
| >30 | 18 (7/39) | 82 (32/39) | 0.88 (0.29-2.62) |  |
| **Lived with someone diagnosed with pulmonary TB** | | | |  |
| Yes | 30 (14/47) | 70 (33/47) | 1.74 (0.88-3.44) |  |
| No | 20 (67/342) | 80 (275/342) | Reference |  |
| Don’t know | 0 (0/3) | 100 (3/3) | undefined |  |
| **Ever used face mask or N 95 respirator** | |  |  |  |
| Yes | 18 (30/164) | 82 (134/164) | 0.78 (0.47-1.29) |  |
| No | 22 (51/228) | 78 (177/228) | Reference |  |

OR=Odds Ratio, CI=Confidence interval.

**Table S2 A comparison of the characteristics of HCWs who had TST and QFT-GIT results and who lost to follow-up.**

*Doctors include interns and residents in training.

| **Demography and exposures** | **HCWs participated in TST incidence study % (n/N)** | **HCWs lost to follow-up TST incidence study**  **% (n/N)** | **P Value** | **HCWs participated in QFT-GIT incidence study % (n/N)** | **HCWs lost to follow-up QFT-GIT incidence study**  **% (n/N)** | **P Value** |
| --- | --- | --- | --- | --- | --- | --- |
| **Location of facilities** | | | | | | |
| Rajshahi | 94 (33/35) | 6 (2/35) | <0.01 |  |  |  |
| Khulna | 81 (39/48) | 19 (9/48) |  |  |  |  |
| Chittagong | 75 (15/20) | 25 (5/20) |  |  |  |  |
| Dhaka | 55 (115/208) | 45 (93/208) |  |  |  |  |
| **Sex** | | | | | | |
| Male | 62 (85/138) | 38 (53/138) | 0.27 | 80 (101/127) | 20 (26/127) | 0.29 |
| Female | 68 (117/173) | 32 (56/173) |  | 85 (110/130) | 15 (20/130) |  |
| **History of BCG vaccination** | | | | | | |
| Yes | 67 (161/239) | 33 (78/239) | 0.31 | 83 (176/213) | 17 (37/213) | 0.89 |
| No | 56 (36/64) | 44 (28/64) |  | 79 (31/39) | 21 (8/39) |  |
| Don't know | 63 (5/8) | 37 (3/8) |  | 80 (4/5) | 20 (1/5) |  |
| **Occupational group** | | | | | | |
| Doctors | 44 (14/32) | 56 (18/32) | 0.07 | 69 (22/32) | 31 (10/32) | 0.25 |
| Nurse | 71 (94/132) | 29 (38/132) |  | 82 (79/96) | 18 (17/96) |  |
| Admin Officer | 68 (39/57) | 32 (18/57) |  | 82 (47/57) | 18 (10/57) |  |
| Laboratory Staff | 69 (11/16) | 31 (5/16) |  | 86 (19/22) | 14 (3/22) |  |
| Support Staff | 59 (44/74) | 41 (30/74) |  | 88 (44/50) | 12 (6/50) |  |
| **Education** |  |  |  |  |  | 0.10 |
| 0 to Primary | 53 (16/30) | 47 (14/30) | <0.01 | 100 (12/12) | 0 (0/12) |  |
| Secondary | 60 (42/70) | 40 (28/70) |  | 82 (46/56) | 18 (10/56) |  |
| Higher Secondary | 76 (103/135) | 24 (32/135) |  | 86 (85/99) | 14 (14/99) |  |
| Honors and above | 54 (41/76) | 46 (35/76) |  | 76 (68/90) | 24 (22/90) |  |
| **Duration (in hours per day) of exposures in pulmonary TB ward** | | | | | | |
| <2 | 64 (87/136) | 36 (49/136) | 0.60 | 73 (52/71) | 27 (19/71) | 0.07 |
| >2 | 61 (76/125) | 39 (49/125) |  | 84 (106/126) | 16 (20/126) |  |
| **Duration (in years) of service as HCWs** | | | | | | |
| < 10 | 66 (130/197) | 34 (67/197) | 0.86 | 78 (119/153) | 22 (34/153) | 0.18 |
| 10-20 | 63 (45/72) | 37 (27/72) |  | 84 (59/70) | 16 (11/70) |  |
| >20 | 64 (27/42) | 36 (15/42) |  | 94 (16/17) | 6 (1/17) |  |
| **Duration (in years) of exposures in pulmonary TB ward** | | | | | | |
| < 10 | 64 (105/165) | 36 (60/165) | 0.87 | 78 (103/132) | 22 (29/132) | 0.20 |
| 10-20 | 60 (38/63) | 40 (25/63) |  | 85 (52/61) | 15 (9/61) |  |
| >20 | 61 (20/33) | 39 (13/33) |  | 94 (15/16) | 6 (1/16) |  |
| **Age of starting work in years** | | | | | | |
| <20 | 58 (21/36) | 42 (15/36) | 0.01 | 84 (26/31) | 16 (5/31) | 0.57 |
| 20-25 | 70 (105/150) | 30 (45/150) |  | 79 (100/127) | 21 (27/127) |  |
| 26-30 | 68 (63/97) | 32 (29/92) |  | 86 (69/80) | 14 (11/80) |  |
| >30 | 41 (13/32) | 59 (19/32) |  | 84 (16/19) | 16 (3/19) |  |
| **Lived someone diagnosed with pulmonary TB at home** | | | | | | |
| Yes | 67 (22/33) | 33 (11/33) | 0.97 | 79 (22/28) | 21 (6/28) | 0.81 |
| No | 65 (178/275) | 35 (97/275) |  | 83 (186/225) | 17 (39/225) |  |
| Don’t know | 67 (2/3) | 33 (1/3) |  | 75 (3/4) | 25 (1/4) |  |
| **Ever used cloth or surgical masks** | | | | | | |
| Yes | 60 (81/134) | 40 (53/134) | 0.15 | 83 (72/87) | 17 (15/87) | 0.84 |
| No | 68 (121/177) | 32 (56/177) |  | 82 (139/170) | 18 (31/170) |  |

# Table S3: Factors associated with discordance between TST and QFT among HCWs in chest diseases hospitals, in Bangladesh

|  | **TST+/QFT+** | **TST-/QFT-** | **TST-/QFT+** | **TST+/QFT-** | **Adjusted OR** | | |
| --- | --- | --- | --- | --- | --- | --- | --- |
|  | **% (n/N)** | **% (n/N)** | **% (n/N)** | **% (n/N)** | **TST+/QFT+ vs**  **TST-/QFT-** | **TST-/QFT+ vs**  **TST+/QFT-** | **TST+/QFT- vs**  **TST-/QFT+** |
| Total | 29 (145/497) | 38 (187/498) | 19 (95/497) | 14 (70/498) | .. | .. | .. |
| **Sex** |  |  |  |  |  |  |  |
| Male | 26 (61/235) | 40 (94/236) | 20 (47/235) | 14 (33/236) | Reference | Reference | Reference |
| Female | 32 (84/262) | 36 (93/262) | 18 (48/262) | 14 (37/262) | 1.40 (0.94-2.10) | 1.01 (0.63-1.61) | 1.00 (0.59-1.68) |
| History of BCG vaccination | | | | | | | |
| No | 29 (25/87) | 37 (32/87) | 26 (23/87) | 8 (7/87) | Reference |  |  |
| Yes | 30 (120/403) | 37 (151/404) | 17 (70/403) | 15 (62/404) | 1.02 (0.60-1.72) | 0.62 (0.35-1.09) | 2.09 (0.91-4.80) |
| Don’t know | 0 (0/7) | 57 (4/7) | 29 (2/7) | 14 (1/7) | undefined | 1.06 (0.18-6.12) | 1.81 (0.19-17.63) |
| **Category of profession** | | | | | | | |
| Doctors* | 16 (9/57) | 40 (23/58) | 28 (16/57) | 16 (9/58) | 0.52 (0.22-1.20) | 1.74 (0.78-3.90) | 1.43 (0.55-3.75) |
| Nurse | 31 (56/180) | 41 (73/180) | 16 (28/180) | 13 (23/180) | 1.07 (0.54-2.14) | 1.02 (0.44-2.37) | 1.22 (0.47-3.15) |
| Admin Officers | 27 (28/103) | 45 (46/103) | 17 (18/103) | 11 (11/103) | Reference | Reference | Reference |
| Lab workers | 26 (9/35) | 43 (15/35) | 11 (4/35) | 20 (7/35) | 0.91 (0.22-1.20) | 0.68 (0.21-2.22) | 2.07 (0.72-5.94) |
| Support Staff | 35 (43/122) | 25 (30/122) | 24 (29/122) | 16 (20/122) | 1.32 (0.70-2.47) | 1.30 (0.62-2.73) | 1.78 (0.75-4.23) |
| **Education** |  |  |  |  |  |  |  |
| 0 to Primary | 36 (16/44) | 18 (8/44) | 36 (16/44) | 9 (4/44) | 1.86 (0.84-4.09) | 1.62 (0.72-3.67) | 0.52 (0.16-1.73) |
| Secondary | 31 (35/114) | 32 (37/114) | 20 (23/114) | 17 (19/114) | 1.60 (0.88-2.90) | 0.81 (0.42-1.59) | 1.16 (0.57-2.37) |
| Higher Secondary | 33 (61/183) | 42 (77/183) | 13 (23/183) | 12 (22/183) | 1.68 (0.99-2.85) | 0.60 (0.32-1.12) | 0.70 (0.36-1.36) |
| Honors and above | 21 (33/156) | 41 (65/157) | 21 (33/156) | 16 (25/157) | Reference | Reference | Reference |
| **Year working in pulmonary TB patient ward** | | | | | | | |
| <10 | 25 (62/245) | 42 (103/245) | 21 (51/245) | 12 (29/245) | Reference | Reference | Reference |
| 10-20 | 34 (42/125) | 33 (41/126) | 18 (22/125) | 16 (20/126) | 1.40 (0.87-2.25) | 0.80 (0.45-1.42) | 1.34 (0.72-2.50) |
| >20 | 38 (18/47) | 19 (9/47) | 28 (13/47) | 15 (7/47) | 1.82 (0.92-3.58) | 1.61 (0.74-3.47) | 1.22 (0.48-3.06) |
| **Hour working in pulmonary TB patient ward** | | | | | | | |
| <2 | 24 (34/139) | 41 (57/139) | 24 (34/139) | 10 (14/139) | Reference | Reference | Reference |
| >2 | 34 (88/257) | 33 (84/257) | 17 (43/257) | 16 (42/257) | 1.47 (0.88-2.48) | 0.63 (0.35-1.12) | 1.92 (0.95-3.89) |
| **Year working as HCWs** | | | | | | | |
| <10 | 25 (67/271) | 42 (115/271) | 19 (51/271) | 14 (38/271) | Reference | Reference | Reference |
| 10-20 | 38 (57/151) | 30 (46/151) | 16 (24/151) | 16 (24/151) | **1.57 (1.01-2.43)** | 0.87 (0.50-1.51) | 1.10 (0.62-1.94) |
| <20 | 39 (21/54) | 17 (9/54) | 30 (16/54) | 15 (8/54) | 1.57 (0.83-3.00) | 2.07 (1.01-4.25) | 1.09 (0.47-2.58) |
| **Age of starting work in years** | | | | | | | |
| <20 | 33 (22/67) | 31 (21/67) | 21 (14/67) | 15 (10/67) | Reference | Reference |  |
| 21-25 | 29 (65/221) | 45 (100/205) | 13 (29/221) | 12 (27/222) | 0.77 (0.42-1.42) | 0.62 (0.30-1.28) | 0.71 (0.31-1.58) |
| 26-30 | 27 (42/154) | 35 (54/154) | 21 (32/154) | 17 (26/154) | 0.74 (0.39-1.40) | 1.08 (0.52-2.24) | 1.02 (0.46-2.31) |
| >=30 | 30 (16/54) | 22 (12/54) | 35 (19/54) | 13 (7/54) | 0.80 (0.37-1.77) | 2.20 (0.96-5.05) | 0.77 (0.27-2.23) |
| **Ever use of face mask/N95 respirators** | | | | | | | |
| Yes | 38 (679/207) | 29 (61/207) | 20 (41/207) | 13 (26/207) | **2.01 (1.34-2.98)** | 1.13 (0.70-1.81) | 0.80 (0.47-1.38) |
| No | 23 (66/290) | 43 (126/291) | 19 (54/290) | 15 (44/291) | Reference | Reference | Reference |
| **Ever lived with pulmonary TB patients** | | | | | | | |
| Yes | 32 (19/59) | 37 (22/59) | 20 (12/59) | 10 (6/59) | 1.12 (0.62-2.02) | 1.00 (0.49-2.07) | 0.65 (0.27-1.61) |
| No | 29 (123/430) | 38 (162/431) | 19 (82/430) | 15 (63/431) | Reference | Reference | Reference |
| Don’t know | 43 (3/7) | 43 (3/7) | 00 (0/7) | 14 (1/7) | 1.88 (0.41-8.62) | undefined | 0.88 (0.10-7.50) |

*Doctors include interns and residents in training.
