# Supplementary material for: Prevalence and incidence of tuberculosis infection among healthcare workers in chest diseases hospitals, Bangladesh: Putting infection control into context
Source: PLoS One. 2023 Sep 27;18(9):e0291484. doi: 10.1371/journal.pone.0291484 (PMC10529546; doi:10.1371/journal.pone.0291484)
Supplement: Attachment — Submitted filename: plos1 Oct2022.docx [file pone.0291484.s002.docx]

Review

PLOS1 – Oct 2022

RE: PLOS1 D22-14779

“Latent TB infection in health care workers in chest diseases hospitals in Bangladesh”

This is an interesting paper describing a cross-sectional survey of health care workers in four hospitals in Bangladesh with a follow up of some (a reasonable proportion) with a repeat TST and or IGRA approximately two years later. Results are interesting in that they indicate an approximate 4% annual risk infection estimated by TST or by QFT. The authors performed two-step tuberculin testing at baseline so the TST conversion rate may be considered reasonably robust although the QFT conversion rate of 4% is certainly within the range of variability previously described for this test, particularly in high incidence settings.

Major Comments:

1. The rate of participation at one hospital was 68% at the largest hospital, but the other three smaller hospitals had a much higher participation rate. Some assessment of potential selection bias is important, for example comparing characteristics of workers who did and did not participate – initially and at the 2 year follow-up.

2. On follow up testing two years later the participation in TST was only 53% at the large hospital compared to 84% at the three smaller hospitals. Follow up for QFT was better at 77%. All of this raises again the problems of selection bias terms of who participated and who did not. At minimum, the characteristics of the workers who did or did not participate in the follow up survey should be provided, even as a supplement table. Selection bias could lead to an over or under-estimate of the conversion rate.

3. Several of the risk factors/ characteristics of workers overlap with each other. For example, type of profession is strongly correlated with level of education. Hence to show both without accounting for the other is not that meaningful. I suggest that the level of education should indicate where doctors, nurses and pharmacists lie because it is really the other workers whose level of education may be more variable that are of interest in this parameter. Similarly, age and years of work are strongly correlated. To avoid this problem of being unable to distinguish the effect of age from the effect of years of work the authors should analyze as two non-overlapping variables: (i) age of starting work at the participating hospital, and (ii) years of work at that hospital. In this way one can distinguish the effect of prior exposure until they were hired at the hospital, or exposure since starting work at the Chest hospitals that explains the high initial prevalence of infection.

4. The analysis of incident TST positivity (ie TST conversion) certain factors should not have changed over the two-year interval and should not be included in the model. For example level of education, having lived with someone with TB, years worked on pulmonary TB wards, and years of work as a HCW. What is more relevant is the exposures in the two years interval - whether they worked on the TB wards in the past two years, and if so how many hours per day. Factors that are relevant to baseline or initial prevalence but are not relevant to the incidence of infection over two years should be included in the model (ie “**have they lived in the last two years** with someone with TB”) which would have been a much more useful question, hence table 3 and table 5 should amended to reflect exposures specifically in the two years since they were tested and negative.

5. A major error, (as far as I can see in the data presented), is in the analysis of the initial TST reactions. Results of the first TST should be analyzed separately from results of the second TST which was to elicit the booster phenomenon. There is good evidence that the “booster phenomenon” is much less specific and much more likely affected by BCG vaccination, non-tuberculosis mycobacteria, as well as remote TB exposure. Hence analyzing initial and second TST together simply introduces misclassification into the exposure of interest which is occupational exposure. I recommend separate analyses as a required reanalysis.

6. I also recommend they drop the comparison of TST and IGRA. This is not novel. And the authors seem to suggest that the IGRA is gold standard for incident infection, when the evidence favours that after baseline two-step TST, it is the TST that is the gold standard for conversion. Obviously this is a matter of debate. Bottom line I don’t think the analysis is all that meaningful nor valuable, hence I suggest dropping table S1 and Figure 2.

Minor points:

7. I recognize that two of the authors are based at CDC and an additional one is also at a US institution. However the study is based in Bangladesh, so I believe the authors should refer to WHO recommendations for use of TST or IGRA, not US recommendations (WHO recommends they can be used interchangeably).

8. Along the same lines, perhaps also the term “latent TB infection” which is now mainly a US term should be dropped in favour of “TB infection” which WHO now uses.
