# Supplementary material for: Prevalence and incidence of tuberculosis infection among healthcare workers in chest diseases hospitals, Bangladesh: Putting infection control into context
Source: PLoS One. 2023 Sep 27;18(9):e0291484. doi: 10.1371/journal.pone.0291484 (PMC10529546; doi:10.1371/journal.pone.0291484)
Supplement: Attachment — Submitted filename: PONE-D-22-14779-Response to reviewers comments.docx [file pone.0291484.s003.docx]

PONE-D-22-14779

To

Desmond Kuupiel, PhD

 Academic Editor

 PLOS ONE

Dear Desmond Kuupiel,

We are thankful to the reviewers for their valuable feedback. We also thank you for allowing us to respond to the comments. Based on the helpful feedback, we revised the manuscript and believe it is more precise, clear, and informative. The following is an itemised list of our specific responses to the reviewers’ comments. We have highlighted the changes in the manuscript as well.

We would appreciate your further review. Please contact me directly with any additional questions or comments. We look forward to hearing from you.

Sincerely

Md. Saiful Islam

Corresponding Author

**Comments:** Journal Requirements:

When submitting your revision, we need you to address these additional requirements.

1. Please ensure that your manuscript meets PLOS ONE’s style requirements, including those for file naming. The PLOS ONE style templates can be found at

https://journals.plos.org/plosone/s/file?id=wjVg/PLOSOne_formatting_sample_main_body.pdf and

<https://journals.plos.org/plosone/s/file?id=ba62/PLOSOne_formatting_sample_title_authors_affiliations.pdf>

**Response:** Thank you. The manuscript and the file names have been updated according to PLOS ONE’s style requirements.

**Comment:** 2. Thank you for stating the following in the Acknowledgments Section of your manuscript:

“We would like to thank all the study participants for their time and respect. This research was funded by the United States Centers for Disease Control and Prevention (CDC), through the cooperative agreement grant number 5U01GH1207. icddr,b acknowledges with gratitude the commitment of CDC to its research efforts. icddr,b is also grateful to the Governments of Bangladesh, Canada, Sweden, and the UK for providing core/unrestricted support. We also acknowledge Arkray Healthcare Pvt Ltd for their support.”

We note that you have provided additional information within the Acknowledgements Section that is not currently declared in your Funding Statement. Please note that funding information should not appear in the Acknowledgments section or other areas of your manuscript. We will only publish funding information present in the Funding Statement section of the online submission form.

Please remove any funding-related text from the manuscript and let us know how you would like to update your Funding Statement. Currently, your Funding Statement reads as follows:

“This research was funded by the United States Centers for Disease Control and Prevention (CDC), through the cooperative agreement grant number 5U01GH1207. Md Saiful Islam and Emily S Gurley has received the funding.

The funders had no role in study design, data collection and analysis, decision to publish, or preparation of the manuscript. The findings and conclusions in this report are those of the authors and do not necessarily represent the official position of CDC.”

Please include your amended statements within your cover letter; we will change the online submission form on your behalf.

**Response:** Thank you. We have checked the acknowledgments sections and removed any funding information from there. It should read, “We would like to thank all the study participants for their time and respect. icddr,b is grateful to the Governments of Bangladesh, Canada, Sweden, and the UK for providing core/unrestricted support. We also acknowledge Arkray Healthcare Pvt Ltd for their support.”

The funding statement is correct.

**Comments:** 3. Please include a separate caption for each figure in your manuscript.

**Response:** Separate caption for each figure has been given.

**Comments:** 4. Please ensure that you refer to Figure 2 in your text as, if accepted, production will need this reference to link the reader to the figure

**Response:** Based on the reviewer’s comments, Figure 2 has been deleted.

**Reviewers’ comments:**

**Reviewer #1: General comments**

**Comments:** The authors present the results of a well-designed study of baseline prevalence of positive TST among HCW working at the 4 largest hospitals with pulmonary units for TB patients and a prospective follow-up for those with negative baseline tests. In addition, IGRA testing was also done at one hospital using QFT Gold In-tube assay (QFT). The comparison of TST and QFT results is largely limited to kappa coefficient and ROC curve.

**Response:** Thank you so much for your appreciation. Regarding the TST and QFT-GIT, a supplementary table S2 has been added, and summary findings are discussed in the manuscript as “Table S3 shows results of the analysis of concordance and discordance between the TST and the QFT-GIT test. QFT-only positive results were significantly associated with the number of years working as HCWs after controlling for sex, age of starting work, history of BCG vaccine, and history of having a household member with pulmonary TB. In contrast, TST-only positive results had no association with the exposures in the model. Both TST and QFT positive results were significantly associated with years working as HCWs after controlling for sex, age of starting work, history of BCG vaccine, and history of having a household member with pulmonary TB’, on page 14, lines 327 -334.

**Comments:** Specific comments

 Methods –**How was the 2-step testing done?**

**Response:** Thank you. On page 7, lines 169-172, we added, “For person whose indurations size was < 10mm in the first-TST, we requested that person for a second test after 14 days from the first test [1]. We took their reading following the same procedure used in the first step. If the size was > 10 mm, the person was considered infected [1, 2].”

**Comments:** There is no mention of how many or what proportion of the individuals were **positive only on the 2nd test**. Were all the initial test results recorded or only those with at least 10 mm of induration? The frequency of “boosting” would be of interest in this setting.

**Response:** All the initial test results were recorded. On page 11, lines 251-256, we added, “Of 665 HCWs who received a two-step TST at baseline, 356 (54%) had a positive result, ranging from 39% to 67% per site. The median induration of the two-step TST was 10mm (IQR 5-13). Of the 356 two-step TST positive, 78% (277/356) were positive by a first-TST (S1). The median induration of the HCWs who were tested positive at the first-TST was 13mm (11mm-15mm) and those who were tested negative at the first-TST was 3mm (0mm-7mm). The median induration of the participants who became TST positive in the second-TST was 16.5mm (15-18)”.

**Comment:** Item that must be addressed:

 There is no mention of obtaining chest radiographs on subjects with positive test results. Were these done or was only **a symptom review don**e? The chest radiograph is considered essential to excluding subclinical TB in the US.

**Response:** Thank you. The primary objective of this research was to estimate the prevalence of latent TB infection among HCWs, and therefore, no chest radiographs was done on HCWs with positive TST and QFT-GIT. However, we added this as a study limitation, “Finally, we did not obtain chest radiographs on HCWs positive with TST or QFT-GIT. We only relied on TB symptoms screening and might have missed cases with active TB” On page 19, lines 449-451.

**Comments:** Results

 Items that must be addressed: Table 2 prevalence of positive TST – footnote iv and v do not indicate adjustment for age.

**Response:** Corrected. Now it reads, “iv Adjusted for age of starting work years, history of BCG vaccine, and lived with someone diagnosed with pulmonary TB.

v Adjusted for age of starting work years, history of BCG vaccine, and lived with someone diagnosed with pulmonary TB” in Table 2.

**Comment:** Table 4 prevalence of positive QFT – footnotes do not include vi. No indication of adjustment for age in footnote iii.

**Response:** Corrected in Table 4. We have revised it as “iii Adjusted for the history of BCG vaccine, age of starting to work in years and lived with someone diagnosed with pulmonary TB.

vi Adjusted for the age of starting to work in years and lived with someone diagnosed with pulmonary TB”.

**Comment:**  Page 15 second sentence states the prevalence of positive TST and QFT were both associated with years of service on pulmonary wards, but this is not indicated in Table 4.

**Response:** The statement is true. In Table 4, HCWs working in pulmonary TB wards >20 years were 2.88 (95% CI 1.14-4.59) times higher at risk of being QFT-GIT positive when compared with HCWs working <10 years in the same wards.

**Comments:** Missing analysis:

 There is a consistent increase in QFT-positivity by age as one expects given the cumulative exposure in the community where they live and work. A more detailed comparison of the age specific variation in concordance and discordance of TST and QFT results by age for the 400-plus HCW with both tests would be of considerable interest. The number and proportion of results that are positive by both, positive for TST alone, QFT alone and negative for both within age categories would be of considerable interest. Those with a positive TST only, tend to be false-positive TST and those with a positive QFT alone tend to be false-positive QFT (except in older individuals) in the context of US HCW but that “tendency” based on follow-up results may not be the same for individuals within this higher-risk population. None of these immunological test results can be interpreted with full confidence of course. Presenting the data on QFT and TST in this setting should include a more detailed analysis and the authors assessment of whether IGRA are useful in this setting.

**Response:** Thank you. We have added a supplementary table, S3 focusing on the concordance and discordance of TST and QFT-GIT. In the text, we said, “S3 shows results of the analysis of concordance and discordance between the TST and the QFT-GIT test. QFT only positive results were significantly associated with number of years working as HCWs after controlling for sex, age of starting to work, history of BCG vaccine, and history of having a household member with pulmonary TB, whereas TST-only positive results had no association with the exposures in the model. Having both TST and QFT positive results were significantly associated with years working as HCWs after controlling for sex, age of starting to work, history of BCG vaccine, and history of having a household member with pulmonary TB”.

**Reviewer #2: RE: PLOS1 D22-14779**

**Comment:** “Latent TB infection in health care workers in chest diseases hospitals in Bangladesh”

 This is an interesting paper describing a cross-sectional survey of health care workers in four hospitals in Bangladesh with a follow up of some (a reasonable proportion) with a repeat TST and or IGRA approximately two years later. Results are interesting in that they indicate an approximate 4% annual risk infection estimated by TST or by QFT. The authors performed two-step tuberculin testing at baseline so the TST conversion rate may be considered reasonably robust although the QFT conversion rate of 4% is certainly within the range of variability previously described for this test, particularly in high incidence settings.

**Response:** Thank you.

**Major Comments:**

**Comments:** 1. The rate of participation at one hospital was 68% at the largest hospital, but the other three smaller hospitals had a much higher participation rate. Some assessment of potential selection bias is important, for example comparing characteristics of workers who did and did not participate – initially and at the 2-year follow-up.

**Response:** We added a supplementary table S2 comparing the characteristics of the participants and non-participants in the follow-up study. In the manuscript, we said, “Table S2 showed that HCWS who were lost to follow up for TST statistically significantly varied by location of hospitals, education, and age of starting to work’ on pages 12-13, lines 285-287.

On page 14, lines 316-19, we added, “Table S2 showed that there was no statistically significant difference in HCWs’ characteristics who participated in the follow-up QFT-GIT and who were lost to follow up”.

In the limitation, we added, “A second limitation was participation attrition; 35% of HCWs were lost to follow up for the repeat TST and 23% for the repeat QFT-GIT due to job transfer, illness, retirement from work, and unwillingness to participate. These participants lost to follow-up could have developed a new infection, and therefore, our study could have inaccurately estimated the true incidence of TST and QFT-GIT positivity. In addition, owing to the loss of follow-up, the incidence analysis was underpowered to detect true differences between groups, particularly by professional category” on page 19, lines 443-449.

We do not have information from HCWs who did not participate in the baseline study, and therefore, it was beyond the scope of the study to compare the characteristics between participants and non-participants at baseline.

**Comment:** 2. On follow up testing two years later the participation in TST was only 53% at the large hospital compared to 84% at the three smaller hospitals. Follow up for QFT was better at 77%. All of this raises again the problems of selection bias terms of who participated and who did not. At minimum, the characteristics of the workers who did or did not participate in the follow up survey should be provided, even as a supplement table. Selection bias could lead to an over or under-estimate of the conversion rate.

**Response:** We added a supplementary table S2 comparing the characteristics of the participants and non-participants in the follow-up study. In the manuscript, we said, “Table S2 showed that HCWS who were lost to follow up for TST statistically significantly varied by location of hospitals, education, and age of starting to work’ on pages 12-13, lines 285-287.

On page 14, lines 317-19, we added, “Table S2 showed that there was no statistically significant difference in HCWs’ characteristics who participated in the follow-up QFT-GIT and who were lost to follow up”.

In the limitation, we added, “A second limitation was participation attrition; 35% of HCWs were lost to follow up for the repeat TST and 23% for the repeat QFT-GIT due to job transfer, illness, retirement from work, and unwillingness to participate. These participants lost to follow-up could have developed a new infection, and therefore, our study could have inaccurately estimated the true incidence of TST and QFT-GIT positivity. In addition, owing to the loss of follow-up, the incidence analysis was underpowered to detect true differences between groups, particularly by professional category” on page 19, lines 443-449.

**Comment:** 3. Several of the risk factors/ characteristics of workers overlap with each other. For example, type of profession is strongly correlated with level of education. Hence to show both without accounting for the other is not that meaningful. I suggest that the level of education should indicate where doctors, nurses and pharmacists lie because it is really the other workers whose level of education may be more variable that are of interest in this parameter.

**Response:** We would like to clarify that we assessed the variance inflation factor (VIF) that measures how the variance of an independent variable is influenced or inflated due to its correlation or interaction with other independent variables in the models, and risk factors with VIF of more than 5 were examined for collinearity and dropped from the models [3]. We did not find any collinearity between education and the category of a profession.

**Comment:** Similarly, age and years of work are strongly correlated. To avoid this problem of being unable to distinguish the effect of age from the effect of years of work the authors should analyse as two non-overlapping variables: (i) age of starting work at the participating hospital, and (ii) years of work at that hospital. In this way one can distinguish the effect of prior exposure until they were hired at the hospital, or exposure since starting work at the Chest hospitals that explains the high initial prevalence of infection.

**Response:** Thank you. Based on your suggestion, we have now used the variable “Age of starting work” instead of age and “years of work in the hospital”.

**Comments:** 4. The analysis of incident TST positivity (ie TST conversion) certain factors should not have changed over the two-year interval and should not be included in the model. For example, level of education, having lived with someone with TB, years worked on pulmonary TB wards, and years of work as a HCW. What is more relevant is the exposures in the two years interval - whether they worked on the TB wards in the past two years, and if so how many hours per day. Factors that are relevant to baseline or initial prevalence but are not relevant to the incidence of infection over two years should be included in the model (ie “have they lived in the last two years with someone with TB”) which would have been a much more useful question, hence table 3 and table 5 should amended to reflect exposures specifically in the two years since they were tested and negative.

**Response:** Thank you. We agree with the reviewer that the two years of exposure could have been more helpful. However, we did not collect the two years exposures separately and therefore have not been used in the model.

**Comment:** 5. A major error (as far as I can see in the data presented), is in the analysis of the initial TST reactions. Results of the first TST should be analysed separately from results of the second TST which was to elicit the booster phenomenon. There is good evidence that the “booster phenomenon” is much less specific and much more likely affected by BCG vaccination, non-tuberculosis mycobacteria, as well as remote TB exposure. Hence analysing initial and second TST together simply introduces misclassification into the exposure of interest which is occupational exposure. I recommend separate analyses as a required reanalysis.

**Response:** Thank you for your concern. We now analysed the results of the first TST separately and added the results as a supplementary table S1.

We want to clarify that the TST analysis and the interpretation have been done following the guidance given by Nayak and Acharjya (2012)[4], and this statement is now added on page 8, lines 187-189. The article Nayak and Acharjya (2012) says regarding booster effect as, “In some persons who are infected with M. tuberculosis, the ability to react to tuberculin may wane over time. When given TST years after infection, these persons may have a false-negative reaction. However, the TST may stimulate the immune system, causing a positive or boosted reaction to subsequent tests. Giving a second TST after an initial negative TST reaction is called two-step testing. When sensitisation to mycobacteria has occurred many years earlier, an initial intradermal injection of tuberculin may produce a negative or weakly positive response due to there being too few sensitised lymphocytes in circulation to produce a significant local response. If the test is repeated, a larger reading may be obtained due to the immune response being ‘recalled ‘or ‘boosted’ by the first test. **The second boosted reading is the correct one – that is, the result that should be used for decision-making or future comparison.** Boosting is maximal if the second test is placed between one and five weeks after the initial test, and it may continue to be observed for up to two years.”

Moreover, two-step TST is recommended to avoid misclassification of subsequent positive TSTs as a TST conversion, indicating recent infection when they are actually a result of boosting[5].

 6. I also recommend they drop the comparison of TST and IGRA. This is not novel. And the authors seem to suggest that the IGRA is gold standard for incident infection, when the evidence favours that after baseline two-step TST, it is the TST that is the gold standard for conversion. Obviously this is a matter of debate. Bottom line I don’t think the analysis is all that meaningful nor valuable, hence I suggest dropping table S1 and Figure 2.

**Response:** Thank you. Based on your suggestion, we have dropped Table S1 and Figure 2.

 Minor points:

**Comments:** 7. I recognise that two of the authors are based at CDC and an additional one is also at a US institution. However, the study is based in Bangladesh, so I believe the authors should refer to WHO recommendations for use of TST or IGRA, not US recommendations (WHO recommends they can be used interchangeably).

**Response:** We have now deleted this from page 5, lines 113-119, “The US Centers for Disease Control and Prevention (CDC) and the Infectious Diseases Society of America/American and Thoracic Society (ATS) clinical guidelines recommend using an IGRA such as QuantiFERON®-TB Gold In-Tube test (QFT-GIT) rather than TST to test adults who are likely to be infected with M. tuberculosis, have a low or medium risk of disease progression, or have a history of BCG vaccination [6]. Guidelines recommend TST when the laboratory capacity required for an IGRA is unavailable [6].”

**Comments:** 8. Along the same lines, perhaps also the term “latent TB infection” which is now mainly a US term should be dropped in favour of “TB infection” which WHO now uses.

**Response:** Many WHO guidelines still use latent TB infection in their policies and guidelines[7]. Therefore, we kept the term “latent TB infection”.

**References:**

1. Lien, L.T., et al., Prevalence and risk factors for tuberculosis infection among hospital workers in Hanoi, Viet Nam. PLoS One, 2009. **4**(8): p. e6798.

2. Anonymous, Procedure for administering, reading and interpreting Mantoux tuberculin skin tests to detect infection with M. tuberculosis 2001.

3. Kwon, Y.S., et al., Factors that Predict Negative Results of QuantiFERON-TB Gold In-Tube Test in Patients with Culture-Confirmed Tuberculosis: A Multicenter Retrospective Cohort Study. PLoS One, 2015. **10**(6): p. e0129792.

4. Nayak, S. and B. Acharjya, Mantoux test and its interpretation. Indian Dermatol Online J, 2012. **3**(1): p. 2-6.

5. Lewinsohn, D.M., et al., Official American Thoracic Society/Infectious Diseases Society of America/Centers for Disease Control and Prevention Clinical Practice Guidelines: Diagnosis of Tuberculosis in Adults and Children. Clinical Infectious Diseases, 2016. **64**(2): p. e1-e33.

6. Lewinsohn, D.M., et al., Official American Thoracic Society/Infectious Diseases Society of America/Centers for Disease Control and Prevention Clinical Practice Guidelines: Diagnosis of Tuberculosis in Adults and Children. Clin Infect Dis, 2017. **64**(2): p. 111-115.

7. World Health Organization, WHO consolidated guidelines on tuberculosis Module 1: Prevention-tuberculosis preventative treatment. 2020: Geneva, Switzerland.
