# Supplementary material for: Prevalence and incidence of tuberculosis infection among healthcare workers in chest diseases hospitals, Bangladesh: Putting infection control into context
Source: PLoS One. 2023 Sep 27;18(9):e0291484. doi: 10.1371/journal.pone.0291484 (PMC10529546; doi:10.1371/journal.pone.0291484)
Supplement: Attachment — Submitted filename: PLOS One review July 2023.docx [file pone.0291484.s004.docx]

PLOS One review July 2023

Comment to the authors re P122-14779R1

I initially reviewed this paper some months ago. I found it had many strengths, particularly the careful performance of two-step tuberculin testing and QuantiFERON testing at baseline, and follow-up among employees at one of the four participating hospitals. Plus the use of questionnaire information to look at risk factors for baseline prevalent positive tests and incident positive tests. By and large, the authors have made most important revisions but there remain two issues that must be addressed.

1. Two-step tuberculin testing

I disagree with the authors in their continued assertion that it is the final result of the two-step test that is the most precise and valuable from an epidemiologic point of view. They cite a paper published in the Indian Journal of Dermatology which, with all due respect to the two authors of that review, provides incorrect information. There is good evidence that boosting (or a positive second TST shortly after a negative first TST) is related to much more than remote TB exposure but also to BCG vaccination and non-tuberculosis mycobacterial exposure, both of which would have been common in this study population. There was also good evidence that the risk of disease is lower in those with a positive second test compared to the first, which doubtless relates to this lack of specificity. They have performed the analysis I suggested, namely factors associated with the first tuberculin test positivity but relegate this to the supplement. I suggest that the primary analysis should be factors associated with a positive first TST, and the factors associated with the positive second test (ie the booster phenomenon) are in the supplement. The analysis of factors associated with any positive test at baseline should be dropped completely. I personally consider this mandatory. Perhaps the co-authors from CDC can suggest other reviews and guidance on the topic of two-step TST and interpretation and management.

2. Past TB disease exclusion

A somewhat more minor point but I think also should be considered strongly by the authors is that the persons with past TB disease were excluded from analyses. This does not really make sense when they are interested in occupational tuberculosis infection. TB disease is the most important manifestation of occupational TB exposure and so these individuals should have been included. If tuberculin testing or QuantiFERON was not done, I would simply assume that they would have had a positive test and classify them as having TB infection. This is only 2% of participants but nevertheless this is an important group and should not be excluded.

Minor points:

1. Exposure in hospitals is not just related to the TB ward or to patients with recognized or diagnosed TB. Of course, in Chest Hospitals all patients who are admitted may be screened for TB disease and so the likelihood that patients on other “non-TB” wards actually have active TB disease that is unrecognized may be lower. However in most general hospitals most TB exposures occur from patients who are admitted for other reasons such as “pneumonia”, have unrecognized TB disease. These individuals are not treated, nor isolated and so represent the greatest hazard to workers. Perhaps the authors can comment on this possibility, had they looked at how many patients with TB disease are in fact admitted to other services or other wards.

2. Latent TB Infection VS TB Infection – The authors prefer to stick with LTBI, given that several co-authors are from CDC;they suggest that WHO documents still refer to LTBI – this is incorrect. Older WHO documents may use the term LTBI but current WHO terminology is TB Infection.

3. Burden VS Incidence – “TB burden” reflects population size and incidence. Hence, countries with very large population size but intermediate incidence may have high burden (e.g., China) but for occupational exposure and risk of infection, I think risk is driven by incidence rather than burden.

4. The authors incorrectly state that IGRAS are not affected by immunosuppression, in fact sensitivity is reduced by immunosuppression, particularly HIV infection, particularly for QuantiFERON, which is the test they have used here. In systematic reviews there is little difference in the sensitivity of QuantiFERON VS TST in immunocompromised populations.

5. They also state that IGRAs are less affected by NTM. There is theoretical evidence that NTM may affect IGRAS less but in reality, there is no actual epidemiological data that I am aware of that shows this. Can the authors provide a citation for a study showing this?
