# Supplementary material for: Prevalence and incidence of tuberculosis infection among healthcare workers in chest diseases hospitals, Bangladesh: Putting infection control into context
Source: PLoS One. 2023 Sep 27;18(9):e0291484. doi: 10.1371/journal.pone.0291484 (PMC10529546; doi:10.1371/journal.pone.0291484)
Supplement: Attachment — Submitted filename: Response to reviewers comments_2_PONE-D-22-14779R1.docx [file pone.0291484.s005.docx]

PONE-D-22-14779R1

To

Frederick Quinn

Academic Editor

 PLOS ONE

Dear Frederick Quinn,

We are thankful to the reviewers for their 2^nd^ round of comments and feedback. We also thank you for allowing us to respond to the comments. Based on the helpful feedback, we revised the manuscript and believe it is more precise, clear, and informative. The following is an itemised list of our specific responses to the reviewers’ comments. We have highlighted the changes in the manuscript as well.

We would appreciate your further review. Please contact me directly with any additional questions or comments. We look forward to hearing from you.

Sincerely

Md. Saiful Islam

Corresponding Author

**Comment**: Reviewer #1: (No Response)

***Response****: Thank you.*

**Comment**: Reviewer #2: Comment to the authors re P122-14779R1
I initially reviewed this paper some months ago. I found it had many strengths, particularly the careful performance of two-step tuberculin testing and QuantiFERON testing at baseline, and follow-up among employees at one of the four participating hospitals. Plus the use of questionnaire information to look at risk factors for baseline prevalent positive tests and incident positive tests. By and large, the authors have made most important revisions but there remain two issues that must be addressed.

***Response****: Thank you for your appreciation.*

**Comment**: 1. Two-step tuberculin testing
I disagree with the authors in their continued assertion that it is the final result of the two-step test that is the most precise and valuable from an epidemiologic point of view. They cite a paper published in the Indian Journal of Dermatology which, with all due respect to the two authors of that review, provides incorrect information. There is good evidence that boosting (or a positive second TST shortly after a negative first TST) is related to much more than remote TB exposure but also to BCG vaccination and non-tuberculosis mycobacterial exposure, both of which would have been common in this study population. There was also good evidence that the risk of disease is lower in those with a positive second test compared to the first, which doubtless relates to this lack of specificity. They have performed the analysis I suggested, namely factors associated with the first tuberculin test positivity but relegate this to the supplement. I suggest that the primary analysis should be factors associated with a positive first TST, and the factors associated with the positive second test (ie the booster phenomenon) are in the supplement. The analysis of factors associated with any positive test at baseline should be dropped completely. I personally consider this mandatory. Perhaps the co-authors from CDC can suggest other reviews and guidance on the topic of two-step TST and interpretation and management.

***Response****: Thank you so much for your valuable input. We have now revised the analysis and used one-step TST as primary analysis. Based on the revised analysis, we have updated the results section. On pages 25-26, lines 595-610, now it reads,*

*On pages 10-11, lines 231-259, we also updated the results section as-*

*“Prevalence of TST Positivity*

*Of 731 HCWs who received a one-step TST at baseline, 296 (40%) had a positive result, ranging from 23% to 46% per site. The median induration of the one-step TST was 8mm (IQR 12). Of the 435 HCWs who were negative at one-step TST, 392 (90%) received two-step TST of whom 21% (81/392) were positive (Table S1). The median induration of the HCWs who were tested positive at the one-step TST was 13mm (IQR10mm-15mm) and those who were tested negative at the first-TST was 3mm (IQR 0mm-7mm). The median induration of the participants who became TST positive in the second-TST was 12mm (IQR11-13). The one-step TST prevalence among HCWs working in Hospital C was 46% (31/68), significantly higher (adjusted odds ratio [aOR]=3.58, 95% CI: 1.61–7.96) than the other hospitals (Table 2).*

*In bivariate analyses, study sites, years worked on pulmonary TB wards, longer duration of stay in pulmonary TB wards per day, and longer duration of service as a HCW were significantly associated with two-step TST positivity at baseline. Age of starting work, sex, previous history of BCG vaccination, and ever having had a household member with PTB were not associated with TST positivity (**Table 2**).* *In the multivariable models, the HCWs at hospital C (aOR=3.58, 95% CI: 1.61–7.96), hospital A (aOR=3.08, 95% CI: 1.37–6.90), and hospital D (aOR=3.23, 95% CI: 1.70–6.12) had higher odds of positive TST results compared with HCWs at Hospital B (**Table 2**). Also, HCWs with a history of working 11–20 years and >20 years on pulmonary TB patients wards had significantly higher odds of TST positivity (aOR=2.01, 95% CI: 1.39–2.93 and aOR=2.09, 95% CI: 1.26–3.45) compared with HCWs working less than 10 years on the same wards (**Table 2). Moreover, HCWs spending more than two hours a day in pulmonary TB wards had significantly higher odds of TST positivity (aOR=1.91, 95% CI: 1.37-2.68). Duration of service as a HCW 11-20 years (aOR 2.09, 95% CI: 1.48-2.97) and >20 years (aOR 2.05, 95% CI: 1.28-3.28) were also associated with higher odds of TST positivity compared with those ≤10 years(**Table 2**)”.*

*We have analysed the two-step TST results separately and added as a supplementary table1 on page 31.*

*On page 7 line 164, we also deleted the sentence, “TST analysis and the interpretation have been done following the guidance given by Nayak and Acharjya (2012)” and removed the citation form the Indian Journal of Dermatology.*

**Comment:** 2. Past TB disease exclusion
A somewhat more minor point but I think also should be considered strongly by the authors is that the persons with past TB disease were excluded from analyses. This does not really make sense when they are interested in occupational tuberculosis infection. TB disease is the most important manifestation of occupational TB exposure and so these individuals should have been included. If tuberculin testing or QuantiFERON was not done, I would simply assume that they would have had a positive test and classify them as having TB infection. This is only 2% of participants but nevertheless this is an important group and should not be excluded.

***Response:*** *Thank you. The HCWs with a history of past TB diseases has been included in the analysis and the tables and the manuscript have been updated based on the revised analysis.*

**Comment**: Minor points: 1. Exposure in hospitals is not just related to the TB ward or to patients with recognized or diagnosed TB. Of course, in Chest Hospitals all patients who are admitted may be screened for TB disease and so the likelihood that patients on other “non-TB” wards actually have active TB disease that is unrecognized may be lower. However, in most general hospitals most TB exposures occur from patients who are admitted for other reasons such as “pneumonia”, have unrecognized TB disease. These individuals are not treated, nor isolated and so represent the greatest hazard to workers. Perhaps the authors can comment on this possibility, had they looked at how many patients with TB disease are in fact admitted to other services or other wards.

***Response:*** *We did not have data on how many patients with TB disease were admitted to what services or wards and therefore it is beyond the scope of the study.*

**Comment:** 2. Latent TB Infection VS TB Infection – The authors prefer to stick with LTBI, given that several co-authors are from CDC;they suggest that WHO documents still refer to LTBI – this is incorrect. Older WHO documents may use the term LTBI but current WHO terminology is TB Infection.

**Response:** Thank you. LTBI has been changed to TB infection (TBI) throughout the manuscript.

**Comments:** 3. Burden VS Incidence – “TB burden” reflects population size and incidence. Hence, countries with very large population size but intermediate incidence may have high burden (e.g., China) but for occupational exposure and risk of infection, I think risk is driven by incidence rather than burden.

***Response:*** *We have deleted the word burden and revised the sentence. Now it reads, “In collaboration with NTP, we sought to estimate the prevalence and incidence of TBI using both IGRA and TST and associated risk factors among HCWs in Bangladeshi chest disease hospitals and identify sub-populations of HCWs at the highest risk of TBI who can be targeted by TB IPC interventions.” On page 5 lines 113-121.*

**Comment**: 4. The authors incorrectly state that IGRAS are not affected by immunosuppression, in fact sensitivity is reduced by immunosuppression, particularly HIV infection, particularly for QuantiFERON, which is the test they have used here. In systematic reviews there is little difference in the sensitivity of QuantiFERON VS TST in immunocompromised populations.

***Response:*** *Thank you. We have revised the sentence. Now, it reads, “IGRAs are performed in vitro and have a number of advantages over TST, including no requirement of return visits and being less affected by exposures to BCG vaccination, or by NTM infection” on page 5 lines 105-107.*

**Comment:** 5. They also state that IGRAs are less affected by NTM. There is theoretical evidence that NTM may affect IGRAS less but in reality, there is no actual epidemiological data that I am aware of that shows this. Can the authors provide a citation for a study showing this?

***Response:*** *Thank you. We have now added this reference to support our statement, “Hermansen, T.S., et al., Non-tuberculous mycobacteria and the performance of interferon gamma release assays in Denmark. PLoS One, 2014.* ***9****(4): p. e93986 on page 5 in 107”*
